# Adherence to iron supplementation during the first year of life infants in Izmir, Turkey

**DOI:** 10.1097/MD.0000000000038926

**Published:** 2024-07-19

**Authors:** Merve Tosyali, Feyza Koç

**Affiliations:** a Division of Social Pediatrics, Department of Pediatrics, Ege University Children’s Hospital, Ege University, Faculty of Medicine, Bornova, Izmir, Turkey.

**Keywords:** infant, iron, supplementation

## Abstract

To determine the prevalence of adherence to iron supplementation and the risk factors for incomplete adherence during the first year of life of infants in Izmir, Turkey. In this cross-sectional study, a total of 511 infants aged 2 to 12 months who presented to the Pediatrics outpatient clinics of Ege University Children’s Hospital were included. Mothers (n = 511) who agreed to participate in the study were interviewed face-to-face and a comprehensive questionnaire including questions about the sociodemographic characteristics of the children and the family, and their adherence to iron supplementation was administered. The data obtained from 471 (92.2%) mothers who used iron supplements for their babies were subjected to further statistical analysis. Analyses were performed with SPSS 25.0. Chi-square test was used for univariate analysis and logistic regression analysis was used to determine the independent factors associated with incomplete adherence to iron supplementation. A total of 511 mothers were surveyed. Among the infants of mothers who participated in the study, 471 (92.2%) were taking iron supplementation. Of the infants who received iron supplementation, 58.3% were given iron supplementation with complete adherence. The percentage of complete adherence with iron supplementation was 35.1% between 2 and 4 months, 66.3% between 5 and 8 months, and 52.4% between 9 to 12 months. In univariate analysis, statistically significant differences were found between complete and incomplete adherence to iron supplementation in terms of infant age, time of birth, family income, maternal education, and maternal employment status (*P* < .001). When the data were analyzed using multivariate analysis, only maternal education level and infant age group were found to be statistically significant independent variables for complete and incomplete adherence to iron supplementation (*P* < .001). In populations with a high prevalence of ID, incomplete adherence to iron supplementation is a serious risk factor for ID/IDA. Although iron supplements are routinely given to infants by the Ministry of Health in Turkey, the prevalence of complete adherence to iron supplementation is low. Therefore, in order to increase the rate of complete adherence to iron supplementation, the iron supplementation status of infants should be reviewed in detail at each health child visit and families should be informed about the importance of supplementation to prevent iron deficiency.

## 1. Introduction

Iron is the major nutrient deficiency among children in underdeveloped countries and iron deficiency anemia (IDA) is the most common cause of anemia in childhood.^[1]^ IDA and iron deficiency (ID) without anemia in infancy are ongoing issues that can have severe and possibly irreversible effects on development, including mental, psychomotor, and growth retardations.^[2,3]^

Children aged <24 months are at the highest risk of iron deficiency in any age group. Iron supplementation is considered to be an appropriate way to prevent iron deficiency during infancy. The World Health Organization (WHO) advises the implementation of iron supplementation in countries where the occurrence of anemia exceeds 40%, as well as in infants who do not consume enough iron through their diet. Additionally, it suggests initiating iron supplementation in premature and low-birth-weight infants during their second month, and in term infants during their 6th month.^[4,5]^ According to the American Academy of Pediatrics (AAP), it is advised to administer iron supplementation to premature babies starting from the first month of life, and to term babies who are exclusively breastfed, from the fourth to sixth month of life.^[6]^ However, many countries have recommended daily iron supplementation during infancy.^[7,8]^

In our country, since 2004, iron supplementation of 2 mg/kg daily for premature infants starting from the second month of life and 10 mg daily for term infants starting from the fourth month of life until at least one year of age, regardless of their nutritional and iron status. The Ministry of Health provides free iron supplements to infants in Family Medicine Centers.^[9–12]^ As part of this program, infants aged 9 to 12 months who receive iron supplementation for at least 5 months undergo routine hemogram evaluation to screen for IDA, but there is no effective follow-up program to assess adherence to iron supplementation.^[10,11]^ In the literature, studies evaluating adherence to iron supplementation are limited.^[13]^

Research shows that iron deficiency persists at high rates in the infant age group, despite a routinely administered free iron supplementation program for infants under 1 year of age in Turkey.^[14,15]^ Incomplete adherence to iron supplementation has been reported to be the main reason for this.^[8,13–15]^ Therefore, this study aimed to determine the prevalence of adherence to iron supplementation and the risk factors for incomplete adherence during the first year of life of infants in Izmir, Turkey.

## 2. Methods

Healthy infants who live in city centers and aged 2 to 12 months admitted to pediatrics Well-Child Care Outpatient Clinics of a tertiary referral hospital, in Izmir, the third largest city in Turkey, were included in the study. In total, 540 mothers who brought their infants under one year of age for routine child health follow-up were interviewed.

Mature infants born at 37 weeks of gestation or later and premature infants born between 34 to 36 weeks of gestation were included in the study; while infants with chronic disease, history of prolonged hospitalization, or acute infection, infants who did not meet the inclusion criteria, and whose parents did not consent to enrollment in the study were excluded. This study was conducted by the principles outlined in the Declaration of Helsinki, 2008, and approved by the local ethics committee. Informed consent was obtained from all parents.

**Figure 1.**
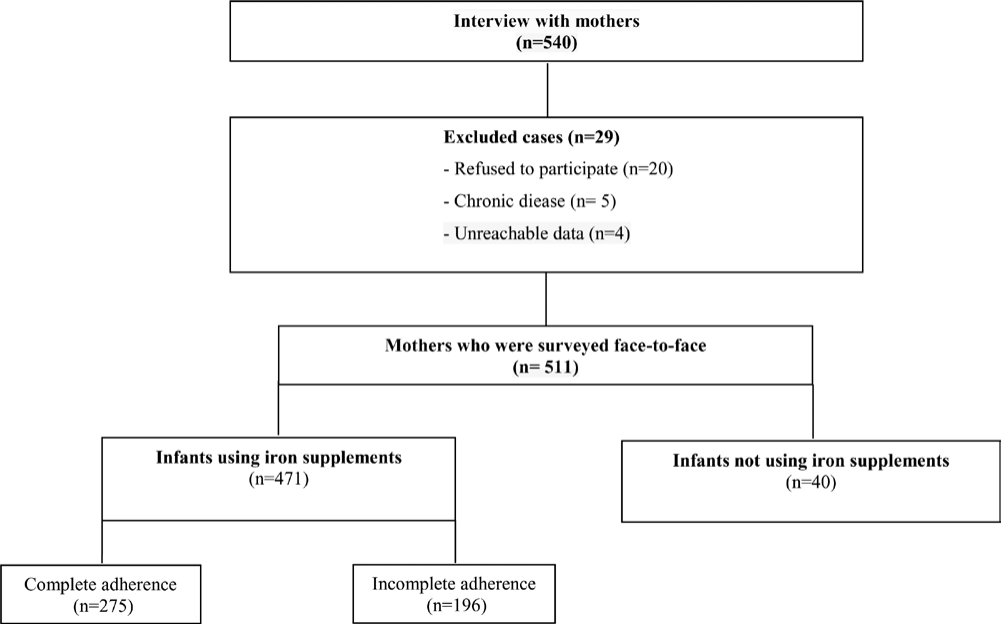
Flow diagram of the recruitment process.

A total of 511 mother-infant pairs were interviewed face-to-face and administered a questionnaire between September 2022 and November 2023. Among the respondents, some infants did not use iron supplements. Therefore, only the data from the 471 mother-infant pairs who used iron supplements were subjected to further statistical analysis. A flow diagram of the recruitment process is shown in Figure 1.

A questionnaire including general characteristics of the subjects such as age, gender, time of birth, family sociodemographic characteristics (economic status, educational level, and employment status of the parents) as well as questions about supplementation practices such as the duration of iron supplementation and adherence to iron supplementation was completed by the mothers. Complete adherence to iron supplementation was defined as daily iron supplementation, as advised by the doctors and as reported by the mother. Only partial (not every day of the week) iron supplementation is defined as incomplete adherence to iron supplementation.

The family’s economic status was determined according to monthly income. The subsistence wage, based on national poverty criteria, is currently $480, which is comparable to the Turkish lira. A family income below this amount was categorized as a low-income family. Monthly income falling between the subsistence wage and 3 times the subsistence wage was categorized as intermediate income. Income above this threshold was categorized as high income.^[16]^

Statistical analysis was performed using the SPSS software (version 25.0; IBM Corp., Armonk, NY, USA). All children were divided into 2 groups for statistical comparisons: complete adherence and incomplete adherence to iron supplementation. The chi-square test was used for univariate analyses to evaluate infant adherence to iron supplementation. Logistic regression analysis was used to find the source of the difference in the variables found to be statistically significant in this analysis and to determine the independent factors associated with incomplete compliance with iron supplementation. A *P*-value < .05 was considered statistically significant.

## 3. Results

In total, 540 mothers were interviewed. Of the mother-infant pairs interviewed, 5.7% did not meet the criteria and were excluded from the study. Of the 511 mothers who were surveyed face-to-face, 92.2% were giving iron supplements to their infants; 7.8% were not using iron supplements. Of the participants, only 471 (92.2%) who were using iron supplements were further statistically analyzed for adherence to iron supplementation (Fig. 1).

**Figure 2.**
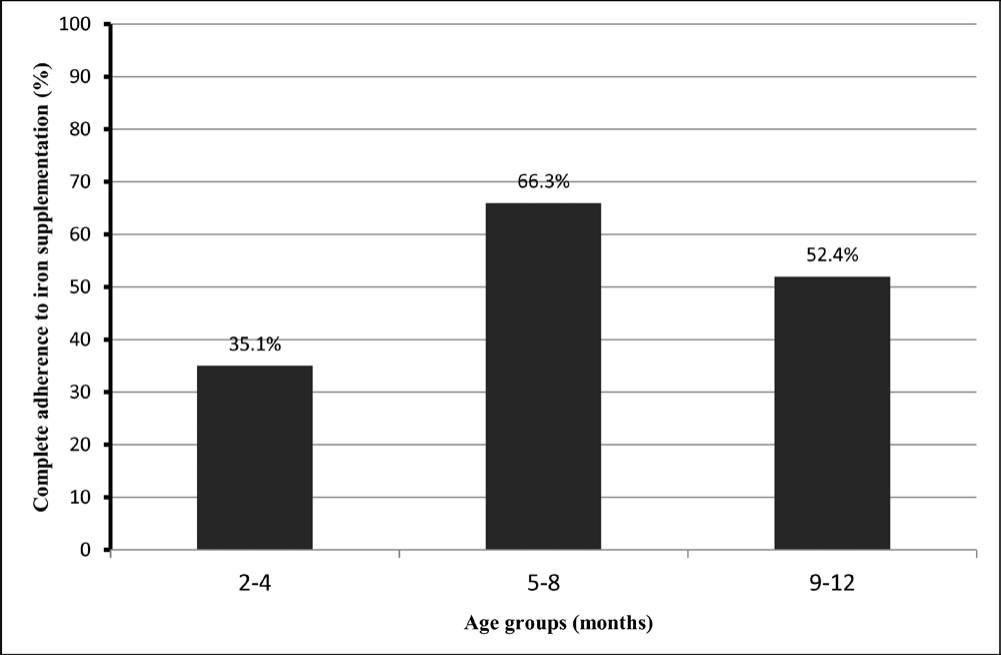
Percentage of complete adherence to iron supplementation by different age groups of infants.

Of the infants, 53.9% were boys and 46.1% were girls. A total of 434 (92.1%) infants had a history of term births, 62.4% of the mothers had a high school education or higher, and only 31.8% were employed (Table 1). 97.9% of the fathers were working (Table 1). 58.4% of participants showed complete adherence to iron supplementation during the first year of life (Fig. 1). The percentage of complete adherence to iron supplementation was 35.1% between 2 and 4 months, 66.3% between 5 and 8 months, and 52.4% between 9 to 12 months (Fig. 2). Among infants using iron supplementation, 41.6% used it with incomplete adherence (Fig. 1). Of the mothers, 15.9% stated that the side effects observed while using iron supplementation (vomiting, bad taste, discoloration of teeth, constipation, diarrhea, etc) were the reason for incomplete adherence to iron supplementation.

**Table 1:** The socio-demographic characteristics of the infants using iron supplements.

	n (%)
**Gender of infants**	
Female	217 (46.1)
Male	254 (53.9)
**Time of birth**	
Mature	434 (92.1)
Premature	37 (7.9)
**Family income**	
Upper–Middle	386 (82)
Lover	85 (18)
**Mother’s education**	
0–8 yrs	177 (37.6)
9–11 yrs	179 (38)
12 or more years	115 (24,4)
**Father’s education**	
0–8 yrs	175 (37,1)
9–11 yrs	151 (32.2)
12 or more years	145 (30.7)
**Maternal employment**	
Unemployed	321 (68.2)
Working	150 (31.8)
**Paternal employment**	
Unemployed	10 (2.1)
Working	461 (97.9)

There was no statistically significant difference between gender and paternal education level in incomplete adherence to iron supplementation. However, in univariate analyses, age groups of infants, time of birth, family income level, maternal education, and maternal employment status were found to be statistically significant independent variables in incomplete adherence to iron supplementation (*P* < .001) (Table 2). The rate of iron supplementation with complete adherence was significantly lower in infants born prematurely than in mature infants (*P* < .001) (Table 2). Families with medium and high family income had higher rates of complete adherence to iron supplementation than those with low family income (*P* < .001) (Table 2). However, when the variables found to be significant were evaluated by multivariate analysis to determine the origin of the difference, it was found that maternal education level and infant age groups were statistically significant independent variables in decreasing adherence to iron supplementation (*P* < .001) (Table 3). Accordingly, the rate of iron supplementation use with complete adherence of infants in the 2 to 4 months group was lower than in other age groups (*P* < .001) (Table 3). When the maternal education status was evaluated, it was found that those with 12 years of education or more had a higher rate of complete adherence to iron supplements (*P* < .001) (Table 3).

**Table 2:** Adherence to iron supplementation according to some demographic characteristics of the study population.

	Adherence to Iron Supplementation, n (%)		
	Complete Adherence	Incomplete Adherence	*P*
**Age groups of infants**			
2–4 mo	13 (35.1)	24 (64.9)	
5–8 mo	165 (66.3)	84 (33.7)	**<.001**
9–12 mo	97 (52.4)	88 (47.6)	
**Time of birth**			
Premature	13 (35.1)	24 (64.9)	**<.001**
Mature	262 (60.4)	172 (39.6)	
**Gender of infants**			
Male	140 (55.1)	114 (44.9)	**.120**
Female	135 (62.2)	82 (37.8)	
**Family income**			
High-Middle	240 (33.3)	146 (66.7)	**<.001**
Low	35 (41.2)	50 (58.8)	
**Maternal education**			
0–8 yrs	76 (42.9)	101 (57.1)	**<.001**
9–11 yrs	111 (62)	68 (38)	
12 or more years	88 (76.5)	27 (23.5)	
**Maternal employment**			
Unemployed	168 (50.8)	153 (46.2)	**<.001**
Employed	107 (71.3)	43 (26.7)	

**Table 3:** Logistic regression analysis to predict risk factors for non to iron supplementation.

Variable	Beta	OR	95% CI	*P-Value*
**Age groups**				
5–8 mo vs 2–4 ms	−1.468	0.23	0.11–0.49	<.001
9–12 mo vs 2–4 ms	−.942	0.39	0.18–0.84	.016
**Maternal education**				
0–8 yrs vs ≥ 12 yrs	1.506	4.51	2.64–7.71	<.001
9–11 yrs vs ≥ 12 yrs	0.659	1.93	1.13–3.31	.016

## 4. Discussion

Iron deficiency remains a major cause of childhood anemia despite national and international iron supplementation initiatives. Infants are particularly vulnerable to ID and IDA, which can negatively impact their health and development, including long-term effects on cognition and behavior.^[1,17–20]^ When an infant develops iron deficiency, addressing it through dietary changes can prevent anemia. However, it may not fix issues with neurodevelopment and long-term cognitive development, because crucial stages of brain growth occur throughout infancy.^[1,7]^ Therefore worries about the negative effects of iron deficiency have resulted in regular utilization of iron supplements to avoid iron deficiency.^[1,6,20,21]^ So, WHO strongly recommends iron supplementation for infants in regions with anemia prevalence above 40%.^[1,5]^ For the same reasons, the AAP also recommends starting prophylactic iron supplementation from the 4th month of life for mature infants and from the 2nd month of life for premature infants.^[6,20–22]^

The “Turkey Like Iron” program has been active in Turkey since 2004.^[10,12]^ Premature infants are given iron supplements every day from the second month of life and mature infants are from the fourth month until the age of one.^[12,13]^ In Turkey, prophylactic iron supplementation is given free of charge to infants aged 4 to 12 months in primary health care facilities. In a study conducted in Turkey in 2013, the effectiveness of the iron supplementation program was evaluated, and it was revealed that the prevalence of anemia in children aged 12 to 23 months was 7.8%.^[9]^ The other studies have reported that ID continues to occur even when infants are given iron supplementation.^[11,20]^ So we thought this might be the cause, our study examined adherence to iron supplementation in infants younger than 12 months. In our study, the prevalence of iron supplementation was 92.2%. However, although the high rates of iron supplement use in our study are similar to the literature, only 58.4% of infants receiving iron supplementation showed complete adherence to iron supplementation. This suggests that a significant proportion of mothers do not adhere to iron supplementation. While the iron supplementation program in Turkey is beneficial, complete adherence to iron supplements remains inadequate. So, increasing the rate of iron supplement use by families with complete adherence is key to reducing the prevalence of iron deficiency in children.

Iron salts are often used for iron supplementation and treatment; unfortunately, their regular use is sometimes interrupted by parents and children due to side effects such as nausea, vomiting, and abdominal pain.^[22–24]^ In our study, 41.6% of the infants who used iron supplements used iron supplements with incomplete adherence, and 15.9% of the mothers stated that side effects such as vomiting, discoloration of the mouth and teeth, constipation, diarrhea, etc seen while using iron supplements were the reason for incomplete adherence to iron supplements. Different studies have shown that iron preparations other than iron salts, such as sucrosomial iron and microencapsulated iron, which have been reported to have fewer GI side effects in recent years, are preferred by some families for this reason.^[25,26]^ Although it is conceivable that the use of these preparations in iron supplementation may increase full compliance with iron supplementation, studies on the efficacy, effectiveness, and safety of sucrosomial or microencapsulated iron preparations prepared in different formulations in the pediatric age group are still limited.^[22,23,26–31]^ Randomized controlled trials are needed to determine whether these preparations are effective in preventing ID in children and what the appropriate dose should be.

In a study conducted in Pakistan in 2001, Ali et al found a positive correlation between education and socioeconomic status and iron intake and a negative correlation with anemia.^[32]^ Another large-sample study showed a clear relationship between maternal education level and the use of prophylactic iron supplements.^[33]^ Incomplete adherence to iron supplementation was not associated with gender and paternal education but was associated with maternal education level and family economic status in our study. The rate of compliance with iron supplementation was significantly higher in infants of mothers with an education level of 12 years or more. So what, the effect of maternal education level on infants’ use of prophylactic iron supplements is generally associated with mothers’ health knowledge and awareness. Mothers with higher levels of education tend to have more knowledge about nutrition and health issues such as IDA. This knowledge enables them to better understand their children’s health needs and make informed decisions about using necessary supplements. Educated mothers may also be more competent in accessing and using health services.

A comprehensive analysis in 2018 showed a significant reduction in anemia rates among children from households with higher socioeconomic status and among children of mothers with higher levels of education.^[34]^ Thus, similar to many previous studies, our study shows that higher maternal education level and higher family economic status are important factors that increase infant complete adherence to iron supplementation.^[32,33]^

In our study, the rate of complete adherence to iron supplementation was lowest between 2 and 4 months and highest between 5 and 8 months infants. Infants aged 2 to 4 months were also premature infants. However, in multivariate analyses, 2 to 4 month-old infants were found to be the age group with the most incomplete adherence to iron supplementation. So, in our study, age and time of birth were important determinants of adherence to iron supplementation. The reasons for incomplete adherence of premature infants to iron supplementation may be the earlier initiation (at 2 mo) of iron supplementation and other conditions that worry the family, such as sucking difficulties, low weight gain, jaundice, etc that may be associated with premature infants in the younger month. However, premature infants are at greater risk for the development of ID/IDA than mature infants. So what is very important is to ensure that premature infants receive iron supplementation with full compliance in the early months.

In our study, we evaluated the factors affecting the complete adherence of infants receiving iron supplements and their parents. Although our study is limited by the small sample size and the fact that it was conducted in a single hospital setting, these results suggest that higher education level and higher family income may be protective factors against iron deficiency by increasing the rate of complete adherence to iron supplementation. Prevention of iron deficiency is crucial to avoid irreversible neurodevelopmental disorders. The fact that, premature infants at high risk for iron deficiency should receive more intensive ID/ IDA follow-up because they do not complete adherence to iron supplementation. Providing detailed information to families at the beginning of iron supplementation by health professionals is of great importance for adherence to iron supplementation. Effective counseling by health professionals on the negative effects of iron deficiency, iron-rich complementary feeding, and the importance of adherence to iron supplementation is essential.

## 5. Conclusion

In a population with a high prevalence of ID, incomplete adherence to iron supplementation is a serious risk factor for ID/IDA. Therefore, it is important to ensure adequate iron intake in the first year of life. Our study results indicated that mothers with higher levels of education and higher family income were more likely to complete adherence to iron supplementation which may be protective factors against iron deficiency. Therefore, policies should be targeted to increase the level of education in society, and policies should be improved to enable families to earn a family income that enables them to live in well-being. The role of healthcare providers is to review infant iron supplementation in detail at every health visit and to inform families about the importance of complete adherence to iron supplementation to prevent iron deficiency. Increasing parental awareness can help increase rates of iron supplement use and complete adherence.

## Acknowledgments

We express our gratitude to the infants and their parents for their involvement in the study.

## Author contributions

**Data curation:** Merve Tosyali, Feyza Koç.

**Formal analysis:** Merve Tosyali, Feyza Koç.

**Investigation:** Merve Tosyali.

**Methodology:** Merve Tosyali, Feyza Koç.

**Project administration:** Feyza Koç.

**Resources:** Feyza Koç, Merve Tosyali.

**Writing – original draft:** Merve Tosyali, Feyza Koç.

**Writing – review & editing:** Merve Tosyali, Feyza Koç.

**Supervision:** Feyza Koç.
